# Distant Temporal Distance and Creative Thinking: The Mediating Role of Promotion Motivation

**DOI:** 10.3389/fpsyg.2020.576835

**Published:** 2020-11-06

**Authors:** Peipei Chen, Jinghuan Zhang, Yiliang Qi

**Affiliations:** ^1^Department of Psychology, Shandong Normal University, Jinan, China; ^2^Zhaogudui Township Government, Jining, China

**Keywords:** distant temporal distance, proximal temporal distance, creative thinking, promotion motivation, undergraduate students

## Abstract

The present study examined the effect of distant temporal distance on creative thinking and the underlying motivation mechanism. We tested our hypotheses in four studies. In Studies 1–4, participants in the distant temporal distance opposed to the proximal temporal distance performed better on a series of creative thinking tasks: the Verbal Divergent Thinking Test (Study 1), the Chinese Remote Associates Test (Study 2), the Toy Design Task (Study 3) and the Ad Evaluation Task (Study 4). Moreover, Studies 2 and 3 found that promotion motivation mediated the beneficial effect of distant temporal distance on the performance of the two creative thinking tasks. In conclusion, distant temporal distance facilitated creative thinking, and promotion motivation mediated this beneficial effect.

## Introduction

In today’s knowledge-based economy, creativity has been emphasized as a key ability needed to obtain success (Florida, 2002). Thus, there has been considerable interest for psychological researchers in identifying the factors that facilitate creativity (Albari et al., 2013). Creativity refers to the ability to produce products that are both novel and useful (Sternberg and Lubart, 1999). Previous studies found that a series of situational factors, such as family and school, played crucial roles in the development of creativity (Pohlman, 1996; Niu and Sternberg, 2003). The current study examined whether an important situational factor – temporal distance – could influence creative thinking. Temporal distance was defined as the extent to which imagined future events or recalled past events deviate from the present in time (Bar-Anan et al., 2006). In addition, to gain a better understanding of the effect of temporal distance on creative thinking, the present study also intended to examine the underlying motivation mechanism through which temporal distance influenced creative thinking.

### Distant Temporal Distance and Creative Thinking

Construal Level Theory (CLT; Liberman and Trope, 1998; Trope and Liberman, 2003) proposes that distant temporal distance fosters abstract thinking. More specifically, when people imagine events in the distant temporal distance, people tend to construct events on the basis of more superordinate and general features that convey central meanings of events (abstract thinking), because of lack of the knowledge about the remote entities, people, events, places, and alternatives.

Theories on creative thoughts indicate that abstract thinking promotes creative cognition (Ward et al., 2004; Friedman and Förster, 2010). Ward et al. (2004), for example, propose that abstract thinking is conducive to the cognitive processing of integration of old and new knowledge, a process that spurs creative thinking. Friedman and Förster (2010) claim that individuals’ abstract constructs enhance their broader conceptual attention, characterized by activating more remote and inaccessible conceptual representations in memory, which is positively related to their creative thinking.

As distant temporal distance enhances abstract thinking, and given that abstract thinking facilitates creative thinking, distant temporal distance might promote creative thinking. Actually, to date, two studies have been conducted to examine the effect of distant temporal distance on creative thinking. Both of them have found that distant temporal distance had a beneficial effect on creative thinking (Förster et al., 2004; Chiu, 2012). However, in the area of creativity research, there exists a common dilemma – a disparity in creativity measurement methods often produces dissimilar results (Hennessey and Amabile, 2010; Martine et al., 2017). Thus, to improve the validity of the result regarding the effect of distant temporal distance on creative thinking, the present study sought to test this effect by using more different types of creative thinking measurement tools. In addition, the present study was also designed to address a shortcoming of the study design of the previous studies. That is, in Förster et al. (2004) and Chiu (2012), all creative thinking tasks have a certain degree of difficulty. Thus, it was possible that the increased creative thinking task performance in distant temporal distance reflected participants’ achievement motivation (defined as the motive that pushes people to engage in tasks with a certain degree of difficulty and to strive to obtain outstanding results in these tasks; Nicholls, 1984) rather than higher levels of creative thinking. To rule out this alternative explanation, the present study (Study 4) used a special creative thinking task which does not support the expression of achievement motivation in order to adduce more robust evidence for the beneficial effect of distant temporal distance.

### The Mediating Role of Promotion Motivation

In addition to examine the impact of distant temporal distance on adolescents’ creative thinking, the current study further sought to investigate the mechanism through which distant temporal distance influences creative thinking. To date, only one study explored the underlying mechanism through which distant temporal distance influenced creative thinking. Specifically, Förster et al. (2004) found that distant temporal distance activated abstract thinking, which in turn promoted creative thinking. Nevertheless, as pointed out by Sternberg and Lubart (1991), not only thinking processing, but also motivation and goals could explain individual differences in creativity. Hence, the present study sought to take into account a motivation mechanism – promotion motivation – to gain a better understanding of why distant temporal distance enhances creative thinking.

### Distant Temporal Distance and Promotion Motivation

According to Regulatory Focus Theory (RFT; Higgins, 1997, 2015), a distinction between two independent yet opposing motivations was made: promotion motivation and prevention motivation. People under a promotion motivation direct their attention, attitudes, and behaviors to achieve their hopes and aspirations, and strive toward advancement and accomplishments. In contrast, people under a prevention motivation direct their attention, attitudes, and behaviors to fulfill their duties and obligations, and strive toward safety and security (Higgins, 2015). This distinction is of critical significance in connecting regulatory focus and temporal distance. That is, with a temporally distant perspective, people are free to imagine what they would like or hope to fulfill. As this temporal resource reduces, however, individuals are obliged to concentrate on what must be achieved, either to sustain life or, less starkly, to fulfill important duties and obligations (Pennington and Roese, 2003). Concisely stated, individuals in distant temporal distance have a greater tendency to become promotion-focused.

Consistent with the perspective that distant temporal distance exerts a beneficial effect on promotion motivation; empirical evidence has also demonstrated this beneficial effect. For instance, Liberman and Trope (1998) reported that when participants made distant-future decisions, they focused more on desirability (promotion-focused concern). In line with this stream of research, Pennington and Roese (2003) proposed that promotion motivation rather than prevention motivation tended to predominate when participants were asked to imagine temporally distant events. Similarly, Mogilner et al. (2008) reported that when ample time remained before purchasing decision-making, people tended to pay more for a product advertised as a means of getting the best potential outcomes (promotion-focused motivation) relative to that advertised as a means of avoiding worse consequences (prevention-focused motivation).

### Promotion Motivation and Creative Thinking

Certain theoretical approaches have been adduced to explain the link between promotion motivation and creative thinking. For example, RFT suggests that the activation of a promotion motivation could be seen as a signaling that the surrounding is prospectively favorable. As a result, an individual with a promotion motivation is more likely to engage in a “riskier” and more heuristic processing style in which original alternatives are desirably and actively sought (Crowe and Higgins, 1997), and, therefore, generates more creative thoughts. Another main theory to comprehend the link regarding promotion motivation and creative thinking is the framework of Derryberry and Tucker (1994). This theory accounted for this link from a perspective of the brain system. More specifically, there exists a brain-based arousal system – the phasic arousal system – which serves to regulate the approach of incentives. When individuals seek incentive cues consciously or unconsciously (promotion-focused motivation), the phasic arousal system is proposed to automatically produce a habituation bias, expanding the scope of conceptual attention and enabling it to flexibly contain novel as well as initially accessible messages, thereby bolstering creative thinking (Friedman and Förster, 2010).

The notion that promotion motivation promotes creative thinking has received substantial empirical support. For example, Mehta and Zhu (2009) reported that when participants were promotion-motivated, they generated more original and novel designs. Similarly, Rook (2014) found that promotion-related appetite, relative to prevention-related aversion, triggered individuals’ creative thinking. Consistent with this, it was reported that promotion-related, rather than prevention-related, anticipatory states favorably affected individuals’ creative problem solving (Friedman and Förster, 2005).

Since distant temporal distance promotes promotion motivation, and seeing that promotion motivation promotes creative thinking, it is possible that promotion motivation would be one route through which distant temporal distance inspires creative thinking. Specifically, we hypothesized that promotion motivation would mediate the beneficial effect of distant temporal distance on creative thinking.

### Overview of the Present Research

In the present study, we tested whether distant temporal distance would promote creative thinking and whether promotion motivation would mediate the beneficial effect of distant temporal distance on creative thinking. Four studies were conducted to address these questions. Specifically, Study 1 tested whether distant temporal distance would enhance creative thinking using a creative thinking task: the Verbal Divergent Thinking Test (VDTT; Runco et al., 2011). Studies 2 and 3 were conducted to provide additional evidence for the results found in Study 1 using two other different types of creative thinking tasks: the Chinese Remote Associates Test (CRAT; Du et al., 2017) and the Toy Design Task (TDT; Moreau and Dahl, 2005), respectively. Study 4, from another perspective, verified the beneficial effect of distant temporal distance on creative thinking by ruling out an alternative explanation concerning the achievement motivation using a special creative task: the Ad Evaluation Task (AET; Zhu and Meyers-Levy, 2007). Furthermore, Studies 2 and 3 tested whether promotion motivation would mediate the effect of distant temporal distance on creative thinking using two different promotion motivation tasks: the Accuracy and Speed Task (AST; Mehta and Zhu 2009) and the Brand Preference Task (BPT; Zhou and Pham, 2004), respectively. Notably, the participants in all four studies differ from each other.

## Study 1

The aim of Study 1 was to examine whether distant temporal distance would enhance creative thinking. We manipulated temporal distance prior to having them complete the creative thinking task. We also added a neutral group in which participants simply finished the creative thinking task without any manipulation. We used the “Travel in Time” task (Förster et al., 2004) to manipulate temporal distance. This procedure has been widely used in many studies to induce distant and proximal temporal distance (Galak et al., 2014; Gauthier and van Wassenhove, 2016). In addition, we adopted the VDTT (Runco et al., 2011) to assess creative thinking, which has proven effective to measure creative thinking by a number of studies (e.g., Zhang et al., 2014; Zhang and Zhang, 2017). We presumed that participants in the distant temporal distance condition would perform better on the VDTT than those in the proximal temporal distance or in the neutral condition.

### Method

#### Participants

A total of 181 law undergraduates (45 males, 136 females; mean age = 18.45, *SD* = 0.57) from Shandong University of Political Science and Law (SUPSL) participated in our survey for a delightful gift and were randomly assigned to the distant temporal distance (*N* = 61), the proximal temporal distance (*N* = 59), or the neutral (*N* = 61) condition. One participant in the distant temporal distance condition was aware of the aims being tested. Thus, we excluded this participant, leaving a final sample of 180 participants. This sample size met the estimated required N of 159 for a one-way ANOVA with three groups to detect a medium effect size (*f* = 0.25) with 80% power, as specified by G*Power software (Faul et al., 2009). All four studies in the current research were approved by the ethics committee of Shandong Normal University.

#### Procedure

On arrival, participants were required to complete several unrelated tasks embedded within a questionnaire packet. To manipulate temporal distance, participants were first instructed to complete the “Travel in Time” task. Specifically, participants in the distant temporal distance condition were asked to imagine their lives 50 years from now for a period of 5 min. Participants in the proximal temporal distance condition were asked to imagine their lives tomorrow for the same period of 5 min. Note that participants in the neutral condition did not receive any particular tasks. Afterward, as a manipulation check for the temporal distance manipulation, the two experimental groups were asked to complete an one-item measure of temporal distance: “How far do you feel the life you imagined is from today?” on a scale from 1 (*very near*) to 7 (*very far*).

Next, all groups completed the VDTT. The task was to ask participants to report as many different uses of a newspaper as one could think of within 4 min. To score these responses, we first eliminated participants’ responses which were not of usefulness and appropriateness. Then, we scored these responses on two dimensions: fluency and originality. Specifically, fluency was scored by taking a simple count of the responses produced by each participant, with repeated answers omitted. Originality was calculated by counting the number of original responses (responses given by less than 5% of the sample were regarded as original). All tasks were timed using a stopwatch by the experimenter.

Finally, participants were probed for suspicion. Specifically, they completed a two-item open-ended scale (Chen et al., 2001): “Do you find anything strange or abnormal regarding the experimental procedure?” and “What do you think is the aim of this study?” All participants neither reported that they found strange or abnormal things nor were aware of the aims being tested except one participant. Participants were then debriefed, thanked and paid.

### Results and Discussion

#### Manipulation Checks

An independent *t* test (distant temporal distance vs. proximal temporal distance) revealed that the temporal distance manipulation was successful, *t* (117) = 31.67, *p* < 0.001, *d* = 5.77. Participants in the distant temporal distance condition felt farther temporal distance (*M* = 5.97, *SD* = 0.92) than those in the proximal temporal distance condition (*M* = 1.41, *SD* = 0.62).

#### VDTT Scores

**Figure 1:**
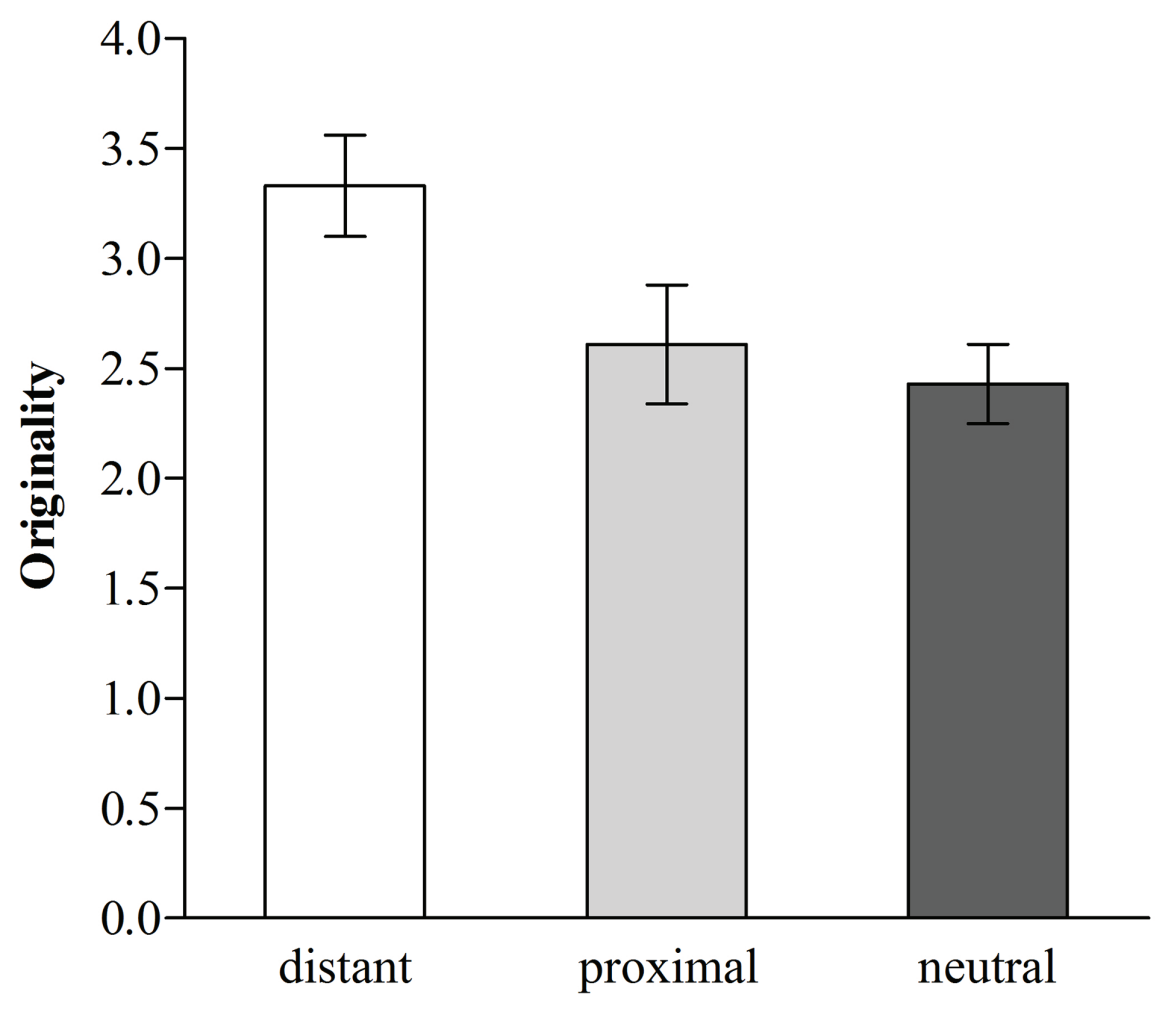
Originality for distant, proximal, and neutral temporal distance (error bars, ±1.00 *SE*; Study 1).

The average fluency score was 7.79 (range = 3–16) and the average originality score was 2.79 (range = 0–7). Thus, we conducted two separate one-way (distant temporal distance vs. proximal temporal distance vs. neutral) ANOVAs in which either the fluency or the originality served as the dependent variable. The ANOVA revealed that the effect of temporal distance on fluency was not significant, *F* (2, 177) = 2.98, *p* > 0.05, *η*^2^ = 0.033. However, the effect of temporal distance on originality was significant, *F* (2, 177) = 4.39, *p* < 0.05, *η*^2^ = 0.047 (see Figure 1). Follow-up Fisher’s least significant difference (LSD) pairwise comparisons (Fisher, 1990) revealed that participants in the distant temporal distance condition demonstrated higher originality scores (*M* = 3.33, *SD* = 1.81) than those in the proximal temporal distance condition [*M* = 2.61, *SD* = 2.04, *d* = 0.37, 95% CI (0.08; 1.37)] or the neutral condition [*M* = 2.43, *SD* = 1.43, *d* = 0.55, 95% CI (0.27; 1.54)], *p* < 0.05 and *p* < 0.01, respectively, which did not differ [*p* > 0.05, 95% CI (−0.46; 0.82)].

The results suggested that distant temporal distance facilitated originality, but exerted no effect on fluency. Because originality is potentially more closely tied theoretically to creative thinking than fluency, it has often solely been used to represent individual’s creative thinking (Sternberg and Lubart, 1999; Dumas and Dunbar, 2014). Thus, we suggested that distant temporal distance facilitated creative thinking.

Interestingly, the result revealed that participants in the proximal temporal distance and neutral conditions had the same VDTT performance, which was consistent with the previous research reporting that participants in the near future and neutral groups had the same creative task performance (Förster et al., 2004). The reason may be attributed to the contention that individuals’ constructs for “tomorrow” and “today” do not differ appreciably in terms of abstractness (Förster et al., 2004).

## Study 2

The first aim of Study 2 was to replicate the result that distant temporal distance promoted creative thinking found in Study 1 using a different creative thinking task: the CRAT (Du et al., 2017), which has been validated and has been used in research with native Chinese participants.

The second aim was to test whether promotion motivation would mediate the beneficial effect of distant temporal distance on creative thinking using a promotion motivation task: the AST (Mehta and Zhu, 2009). This kind of task has proven productive to measure promotion motivation in many previous studies (e.g., Förster et al., 2003; Bullens et al., 2013) with satisfactory reliability and validity. The task was to ask people to indicate whether they focused on speed or accuracy on a three-item scale, and the extent to which participants focused on speed vs. accuracy represented their promotion motivation scores. The logic was that higher levels of promotion motivation activated people’s risky processing (Derryberry and Tucker, 1994; Friedman and Förster, 2002), which promoted them to be concerned with “speed”; lower levels of promotion motivation, instead, activated people’s prudent and cautious processing (Friedman and Förster, 2002), which enabled them to pay more attention to “mistakes” or “accuracy.” We predicted that distant temporal distance would facilitate CRAT scores, and promotion motivation would mediate the beneficial effect of distant temporal distance on CRAT scores.

### Method

#### Participants

A total of 163 law undergraduates, who came from SUPSL (78 males, 85 females; mean age = 18.58, *SD* = 0.68), participated in this study for a delightful gift. They were randomly assigned to the distant temporal distance (*N* = 53), proximal temporal distance (*N* = 54), or neutral (*N* = 56) condition. No participant was aware of the aims being tested and thus no participant was excluded. This sample size met the estimated required N of 159 for a one-way ANOVA with three groups to detect a medium effect size (*f* = 0.25) with 80% power (Faul et al., 2009).

#### Procedure

On arrival, participants were required to finish certain unrelated tasks embedded within a questionnaire packet. To manipulate temporal distance, the two experimental groups were first instructed to complete the “Travel in Time” task used in Study 1. Afterward, as a manipulation check for the temporal distance manipulation, they then completed a one-item measure of temporal distance (see Study 1).

Next, all three groups (the two experimental and neutral group) received the CRAT. The test contains 15 items, each item consisting of three Chinese characters. Participants were asked to come up with the fourth Chinese character [e.g., “动” (move)] that can combine with the three characters [e.g., “词” (word), “运” (transport), and “带” (belt)] to form three two-character phrases [e.g., “动词” (verb), “运动” (exercise), and “带动” (drive)]. Fifteen minutes were given for the CRAT. We calculated CRAT scores by adding up the number of the correct answers produced by each participant. After finishing the CRAT, the participants were asked to indicate their motivation as they were completing the CRAT on a three-item 7-point scale: “I concentrated on finishing the task as quickly as possible.” (1 = *completely disagree* and 7 = *completely agree*), “I paid more attention to avoiding making mistakes.” (1 = *completely disagree* and 7 = *completely agree*) and “I paid more attention to accuracy than speed.” (1 = *completely disagree* and 7 = *completely agree*). Promotion motivation index was created by first reversely coding the latter two questions, and then averaging the three items scores. All tasks were timed with a stopwatch by the experimenter.

Finally, participants completed the same two-item open-ended suspicion probe used in Study 1. None of the participants reported that they found strange or abnormal things or was aware of the aims being tested. Participants were then debriefed, thanked, and paid.

### Results and Discussion

#### Manipulation Check

An independent *t* test (distant temporal distance vs. proximal temporal distance) revealed that the temporal distance manipulation was successful, *t* (105) = 30.55, *p* < 0.001, *d* = 5.85. Participants in the distant temporal distance condition felt farther temporal distance (*M* = 6.28, *SD* = 0.91) than those in the proximal temporal distance condition (*M* = 1.89, *SD* = 0.54).

#### CRAT Scores

**Figure 2:**
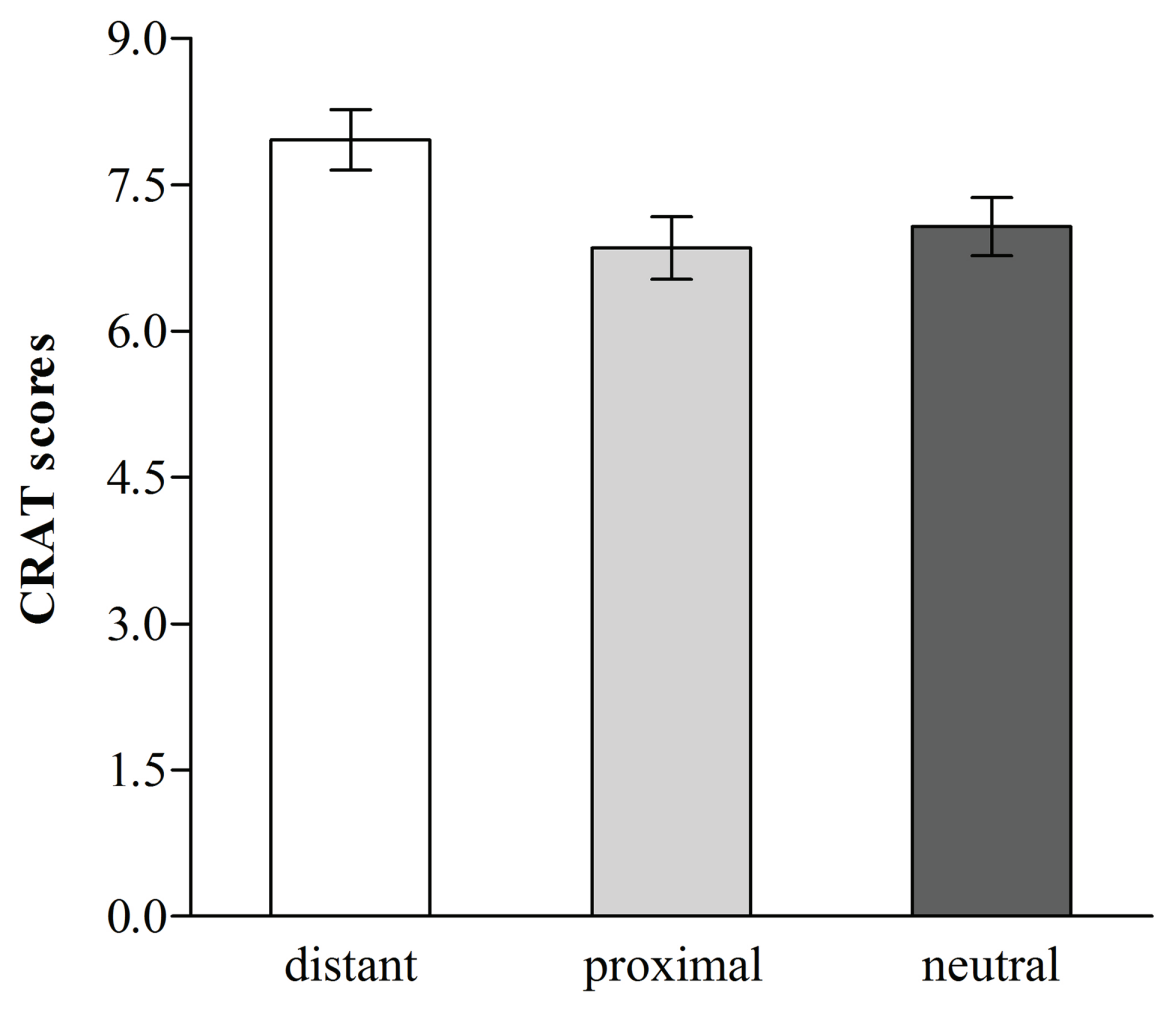
Chinese Remote Associates Test (CRAT) scores for distant, proximal, and neutral temporal distance (error bars, ±1.00 *SE*; Study 2).

The average CRAT score was 7.29 (range = 2–14), and we conducted a one-way (distant temporal distance vs. proximal temporal distance vs. neutral) ANOVA in which the CRAT scores served as the dependent variable. The ANOVA revealed that the effect of temporal distance on CRAT scores was significant, *F* (2, 160) = 3.57, *p* < 0.05, *η*^2^ = 0.043 (see Figure 2). Follow-up Fisher’s LSD pairwise comparisons (Fisher, 1990) found that participants in the distant temporal distance condition produced more correct answers (*M* = 7.96, *SD* = 2.28) than those in the proximal temporal distance [*M* = 6.85, *SD* = 2.32, *d* = 0.48, 95% CI (0.24; 1.98)] or neutral condition [*M* = 7.07, *SD* = 2.23, *d* = 0.40, 95% CI (0.03; 1.75)], *ps* < 0.05, which did not differ [*p* > 0.05, 95% CI (−1.08; 0.64)].

**Figure 3:**
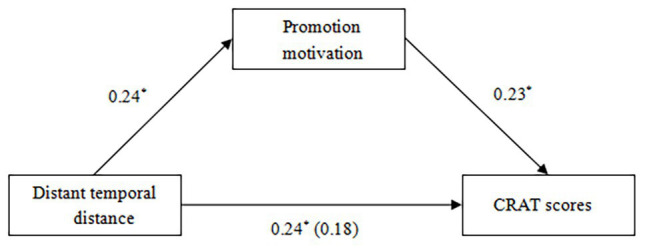
The mediating effect of promotion motivation on the relationship between distant temporal distance and CRAT scores (Study 2). ^*^p < 0.05.

Consistent with Study 1, the findings revealed that distant temporal distance facilitated creative thinking (indicated by CRAT scores). Next, we further tested whether promotion motivation would mediate the relationship between distant temporal distance (1 = the proximal temporal distance condition and 2 = the distant temporal distance condition) and CRAT scores. The correlation analyses revealed that distant temporal distance was positively correlated with promotion motivation (*r* = 0.24, *p* < 0.05) and CRAT scores (*r* = 0.24, *p* < 0.05), and promotion motivation was positively correlated with CRAT scores (*r* = 0.27, *p* < 0.01). Based on these correlation results, three linear regression analyses were then conducted (Hayes, 2013). The first regression analysis revealed that distant temporal distance significantly predicted CRAT scores, *B* = 1.11, *b* = 0.24, *SE* = 0.44, *t* = 2.50, *p* < 0.05, 95% CI [0.23; 1.99]. The second regression analysis revealed that distant temporal distance significantly predicted promotion motivation, *B* = 0.49, *b* = 0.24, *SE* = 0.20, *t* = 2.48, *p* < 0.05, 95% CI (0.10; 0.88). The third regression analysis revealed that promotion motivation significantly predicted CRAT scores, *B* = 0.52, *b* = 0.23, *SE* = 0.22, *t* = 2.38, *p* < 0.05, 95% CI (0.09; 0.94), and that when accounting for this relationship, the effect of distant temporal distance on CRAT scores was not significant, *B* = 0.86, *b* = 0.18, *SE* = 0.45, *t* = 1.92, *p* > 0.05, 95% CI (−0.03; 1.75). The results of the linear regression analyses suggested that promotion motivation mediated the beneficial effect of distant temporal distance effect on CRAT scores. We continued to test the significance of this mediation effect using the Monte Carlo method (MacKinnon et al., 2004). The result showed that 95% CI for this mediation effect was [0.01, 0.63], not including zero, demonstrated that the mediating effect of promotion motivation on the link between distant temporal distance and CRAT scores was significant (see Figure 3).

Overall, Study 2 replicated the result of Study 1 that distant temporal distance facilitated creative thinking. Moreover, the result of Study 2 further found that promotion motivation mediated the beneficial effect of distant temporal distance on creative thinking.

## Study 3

Study 3 was designed with two main objectives in mind. First, we conducted Study 3 to provide additional evidence for the beneficial effect of distant temporal distance on creative thinking observed in Studies 1 and 2 by adopting a new creative thinking task: the TDT (Moreau and Dahl, 2005). This kind of task is one of the most widely used tasks to effectively measure creative thinking (e.g., Chiu, 2012). Second, Study 3 was to replicate the mediating role of promotion motivation through which distant temporal distance promoted creative thinking found in Study 2 by using a different promotion motivation task: the BPT (Zhou and Pham, 2004). Previous study employing this task to measure promotion motivation proved good reliability and validity (e.g., Zhu, 2007; Mehta and Zhu, 2009). The task was to ask participants to indicate their preferences on three pair of brands on a 7-point scale (1 = *prefer brand A* and 7 = *prefer brand B*). Within each pair, brand A (a toothbrush that functions in cavity prevention) represents a negative goal people attempt to avoid, whereas brand B (e.g., a toothbrush that functions in tooth whitening) represents a positive goal individuals attempt to approach. Promotion motivation index was created by the extent to which people prefer brand B. The logic was that people with high levels of promotion motivation preferred brands which provided promotion benefits such as tooth whitening, rather than brands that offered prevention benefits such as cavity prevention. Similar to Study 2, we predicted that distant temporal distance would facilitate individual’s TDT scores and promotion motivation would mediate this beneficial effect.

### Method

#### Participants

A total of 185 law undergraduates (59 males, 126 females; mean age = 18.92, SD = 0.91) from SUPSL participated in this study for a delightful gift and were randomly assigned to the distant temporal distance (*N* = 62), proximal temporal distance (*N* = 62), or neutral (*N* = 61) condition. No participant was aware of the aims being tested and thus no participant was excluded. This sample size met the estimated required *N* of 159 for a one-way ANOVA with three groups to detect a medium effect size (*f* = 0.25) with 80% power (Faul et al., 2009).

#### Procedure

Study 3 was conducted in two stages. In stage 1, participants were required to engage in several unrelated tasks embedded within a questionnaire packet. Afterward, the two experimental groups completed the temporal distance manipulation and the one-item manipulation check measure used in Studies 1 and 2. Next, all three groups completed the TDT. Specially, participants were asked to read the following guidelines and to complete the TDT according to the guidelines, “This is a product design study, please select any 5 parts from the drawings of 20 different parts below to design a toy a child between the ages of 5 and 16 could play with. You should circle the selected five parts and draw your toy design on a blank sheet of paper provided to you (the drawings were showed in the Supplementary Material). Each part is allowed to be used only once, and nonselected parts are not permitted.” After finishing the TDT, participants continued to complete the BPT. Under this task, participants were presented with descriptions of three pairs of brands and were instructed to indicate their preferences on a 7-point scale (1 = *prefer brand A* and 7 = *prefer brand B*). Finally, participants completed the same two-item open-ended suspicion probe employed in Studies 1 and 2. None of the participants reported that they found any strange or abnormal things or was aware of the aims being tested. Participants were then debriefed, thanked, and paid.

In stage 2, we recruited four judges, from the same population, to evaluate each toy design on originality/novelty with the item, “Overall, the design is original and novel,” on a 7-point scale (1 = *not original and novel* and 7 = *very original and novel*). These four judges were all professors or associate professors of design from Shandong Normal University. All of them had extensive training and experience in consumer product design. The reliability of the judges’ ratings was good (*α* > 0.90).

### Results and Discussion

#### Manipulation Check

An independent *t* test (distant temporal distance vs. proximal temporal distance) revealed that the temporal distance manipulation was successful, *t* (122) = 37.06, *p* < 0.001, *d* = 6.68. Participants in the distant temporal distance condition felt farther temporal distance (*M* = 6.46, *SD* = 0.74) than those in the proximal temporal distance condition (*M* = 1.68, *SD* = 0.69).

#### Originality/Novelty

**Figure 4:**
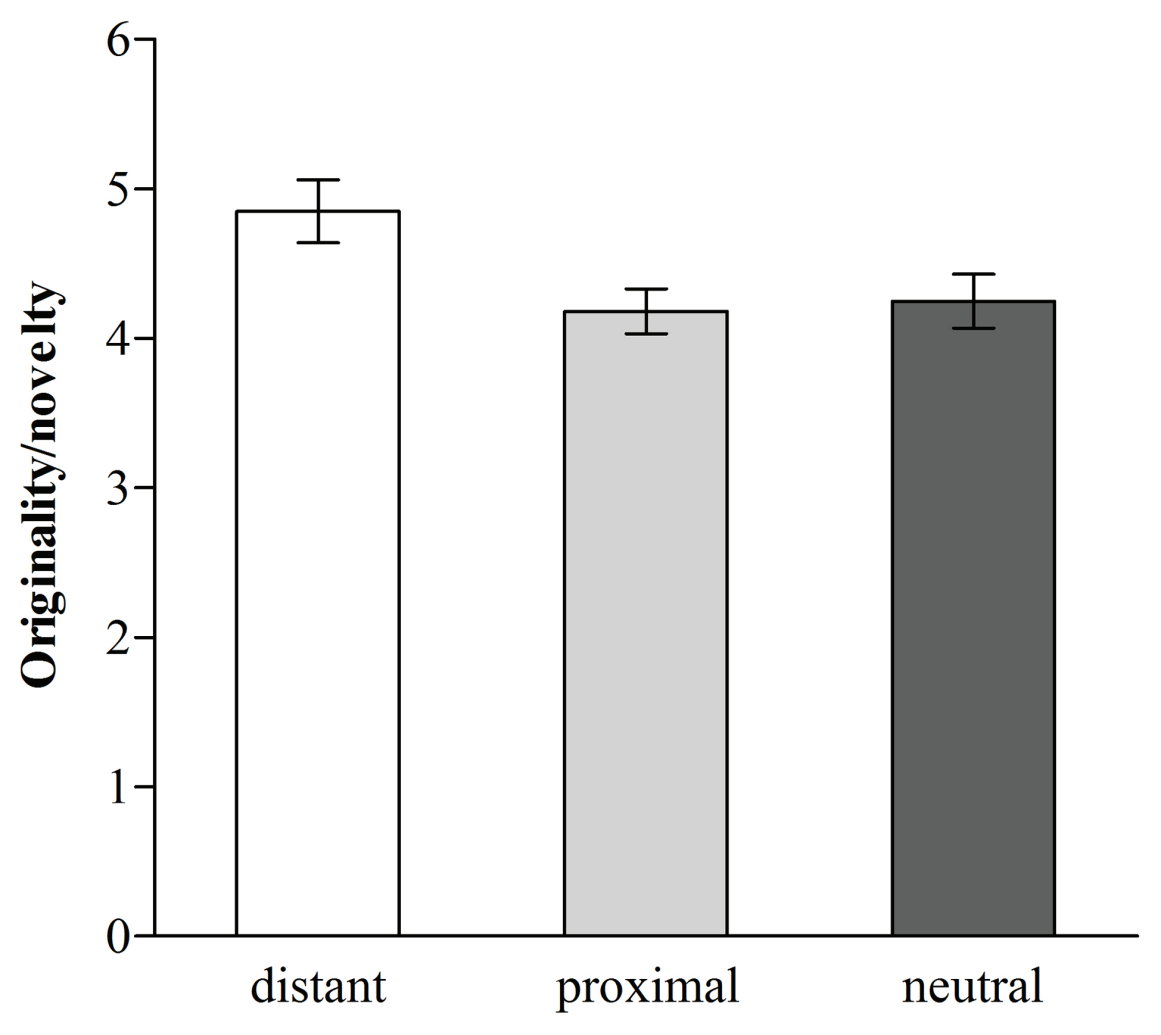
Originality/novelty for distant, proximal, and neutral temporal distance (error bars, ±1.00 *SE*; Study 3).

**Figure 5:**
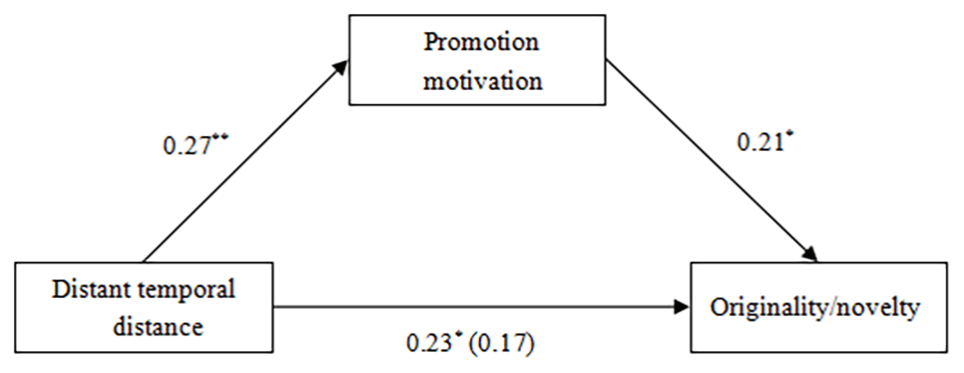
The mediating effect of promotion motivation on the relationship between distant temporal distance and originality/novelty (Study 3). ^*^p < 0.05 and ^**^p < 0.01.

The average originality/novelty score was 4.43 (range = 1–7), and we applied a one-way (distant temporal distance vs. proximal temporal distance vs. neutral) ANOVA in which originality/novelty served as the dependent variable. The ANOVA revealed that the effect of temporal distance on originality/novelty was significant, *F* (2, 182) = 4.02, *p* < 0.05, *η*^2^ = 0.042 (see Figure 4). Follow-up Fisher’s LSD pairwise comparisons (Fisher, 1990) revealed that participants in the distant temporal distance condition (*M* = 4.85, *SD* = 1.69) designed more original and novel toys than those in the proximal temporal distance [*M* = 4.18, *SD* = 1.20, *d* = 0.46, 95% CI (0.16; 1.19)] or neutral condition [*M* = 4.25, *SD* = 1.42, *d =* 0.38, 95% CI (0.09; 1.12)], *ps* < 0.05, which did not differ [*p* > 0.05, 95% CI (−0.59; 0.44)]. The results revealed that distant temporal distance facilitated creative thinking (indicated by originality/novelty). Next, we further tested whether promotion motivation would mediate the relationship between distant temporal distance (1 = the proximal temporal distance condition and 2 = the distant temporal distance condition) and originality/novelty. The correlation analyses revealed that distant temporal distance was positively correlated with promotion motivation (*r* = 0.27, *p* < 0.01) and originality/novelty (*r* = 0.23, *p* < 0.05), and promotion motivation was positively correlated with originality/novelty (*r* = 0.26, *p* < 0.01). Based on these correlation results, three linear regression analyses were then conducted (Hayes, 2013). The first regression revealed that distant temporal distance significantly predicted originality/novelty, *B* = 0.67, *b* = 0.23, *SE* = 0.26, *t* = 2.56, *p* < 0.05, 95% CI (0.15; 1.19). The second regression revealed that distant temporal distance significantly predicted promotion motivation, *B* = 0.81, *b* = 0.27, *SE* = 0.26, *t* = 3.09, *p* < 0.01, 95% CI (0.29; 1.32). The third regression revealed that promotion motivation significantly predicted originality/novelty, *B* = 0.21, *b* = 0.21, *SE* = 0.09, *t* = 2.38, *p* < 0.05, 95% CI (0.04; 0.39), and when accounting for this effect, the effect of distant temporal distance on originality/novelty was not significant, *B* = 0.50, *b* = 0.17, *SE* = 0.27, *t* = 1.87, *p* > 0.05, 95% CI (−0.03; 1.03). The results of linear regression analyses suggested that promotion motivation mediated the beneficial effect of distant temporal distance on originality/novelty, and the mediating effect was also significant [95% CI (0.01; 0.40)] see Figure 5.

Study 3 replicated the results of Studies 1 and 2 that distant temporal distance facilitated individual’s creative thinking and promotion motivation mediated this beneficial effect.

## Study 4

Study 4, from another perspective, verified the beneficial effect of distant temporal distance on creative thinking through ruling out an alternative explanation concerning the achievement motivation using a special creative thinking task: the AET (Zhu and Meyers-Levy, 2007). Specially, there could be an alternative explanation for the beneficial effect of distant temporal distance on creative thinking found in Studies 1, 2, and 3. That is, instead of high levels of creative thinking, the enhanced performance of creative thinking task in the distant temporal distance condition may represent participants’ motivation to succeed in a relatively difficult task. The question that hence remained was whether our hypothesis would be supported in a creative thinking task that does not facilitate the expression of such motivation. To address this question, we conducted Study 4 to examine the effect of distant temporal distance on creative thinking using a special creative thinking task: the AET (Zhu and Meyers-Levy, 2007). The AET was suitable because evaluating an ad was hardly an endeavor one would feel proud of doing well in. To better guarantee the effectiveness of the AET to measure creative thinking, a pretest was also conducted in the present study to evaluate the AET’s cross-validation with other measures of creative thinking.

As a secondary objective, we also tested whether the observed effects were driven by different moods induced by distant vs. proximal temporal distance, since mood influenced creative thinking (Isen et al., 1987; Chirico et al., 2018).

### Method

#### Participants

A total of 165 law undergraduates (71 males, 94 females; mean age = 18.92, *SD* = 0.81) from SUPSL were recruited for the study for a delightful gift. They were randomly assigned to the distant temporal distance (*N* = 55), proximal temporal distance (*N* = 55), or neutral (*N* = 55) condition. No participant was aware of the aims being tested and thus no participant was excluded. This sample size met the estimated required N of 159 for a one-way ANOVA with three groups to detect a medium effect size (*f* = 0.25) with 80% power (Faul et al., 2009).

#### Procedure

On arrival, participants were required to complete several tasks embedded within a questionnaire packet. Afterwards, the two experimental groups completed the same temporal distance manipulation and the same one-item manipulation check measure used in Studies 1 through 3. Next, all groups were asked to report their current moods on a one-item 7-point scale: “How do you feel right now?” (1 = *very bad* and 9 = *very good*). All groups then completed the AET. More specifically, participants were presented with a camera ad which featured a camera image in the middle surrounded by rather ambiguously related associations (the ad picture was showed in the Supplementary Material), and were instructed to evaluate this ad on a three-item 7-point scale: “To what extent do you think the ad appeals to you?” (1 = *not appeal* and 7 = *very appeal*), “To what extent do you think the ad is favorable?” (1 = *not favorable* and 7 = *very favorable*) and “To what extent do you think the ad is effective” (1 = *not effective* and 7 = *very effective*). Responses to the three items were averaged to form an overall score, which represented individuals’ creative thinking. The logic was that higher levels of creative thinking can help participants connect all the ambiguously related associations to a camera-related theme, which resulted in more favorable evaluations, while participants with lower levels of creative thinking would feel that the ad was disorder and unreasonable, thus leading to more negative evaluations. With the AET completed, participants’ current moods were checked again. Finally, participants completed the same two-item open-ended suspicion probe used in Studies 1 through 3. None of the participants reported any strange or abnormal things or was aware of the aims being tested. Participants were then debriefed, thanked and paid.

#### Pretest

A separate sample of 128 economics undergraduates (39 males, 89 females; mean age = 19.64, SD = 0.90) from SUPSL were recruited. They were asked to complete four creative thinking tasks – the VDTT, the CRAT, the TDT, and the AET – whose order was counterbalanced across participants.

### Results and Discussion

#### Cross-Validation of the AET

We first examined the cross-validation of the AET using the pretest data. The results revealed that the AET scores showed positive strong correlations with originality scores of the VDTT (*r* = 0.34, *p* < 0.001), the CRAT scores (*r* = 0.45, *p* < 0.001), and the TDT scores (*r* = 0.44, *p* < 0.001), demonstrating that the AET had good cross-validation.

#### Manipulation Check

An independent *t* test (distant temporal distance vs. proximal temporal distance) revealed that the temporal distance manipulation was successful, *t* (108) = 52.14, *p* < 0.001, *d* = 9.89. Participants in the distant temporal distance condition felt farther temporal distance (*M* = 6.62, *SD* = 0.53) than those in the proximal temporal distance condition (*M* = 1.71, *SD* = 0.46).

#### AET Scores

**Figure 6:**
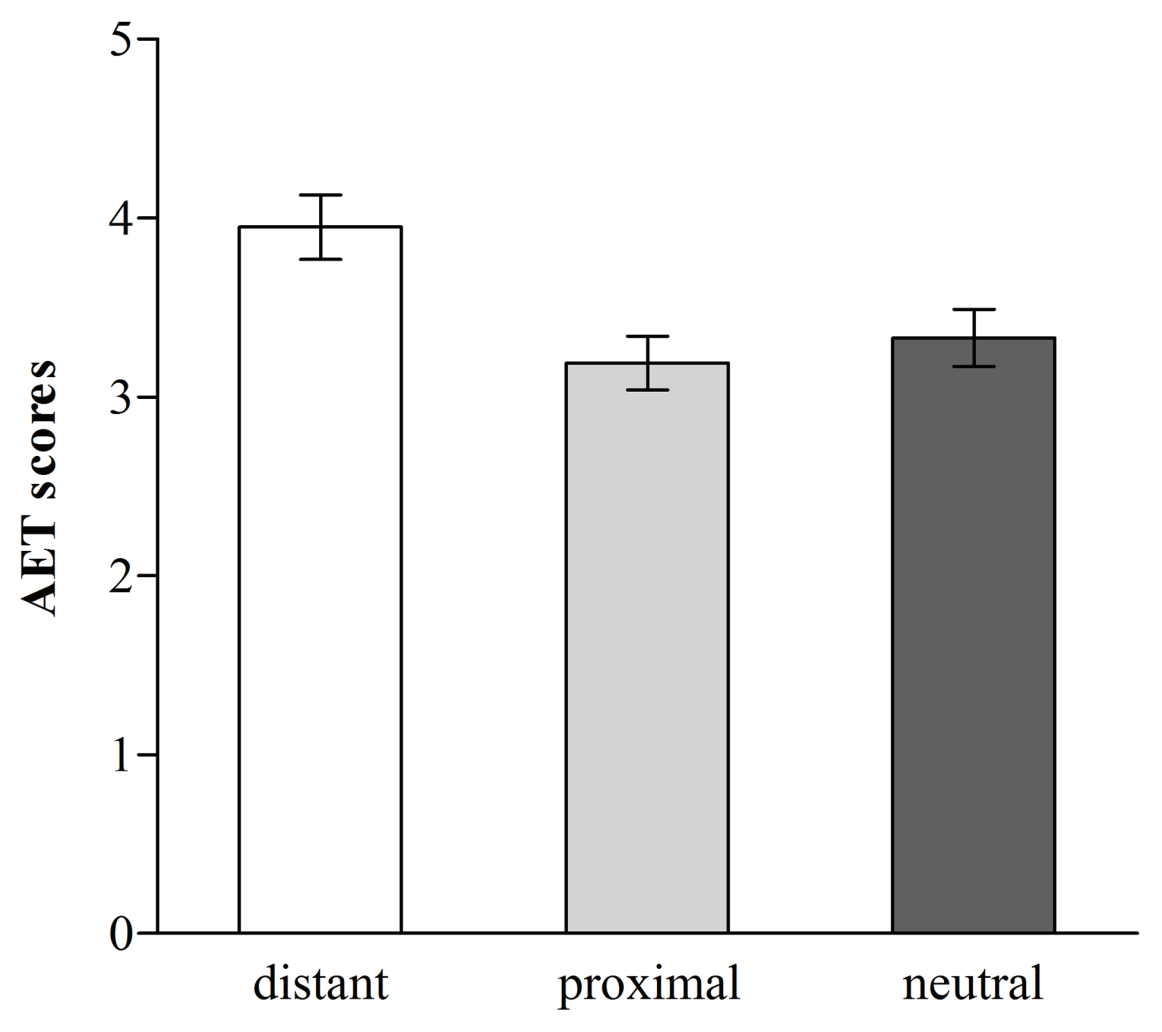
Ad Evaluation Task (AET) scores for distant, proximal, and neutral temporal distance (error bars, ±1.00 *SE*; Study 4).

The average AET score was 3.49 (range = 1–6.67), and we applied a one-way (distant temporal distance vs. proximal temporal distance vs. neutral) ANOVA in which AET scores served as the dependent variable. The ANOVA revealed that the effect of temporal distance on AET scores was significant, *F* (2, 162) = 5.72, *p* < 0.01, *η*^2^ = 0.066 (see Figure 6). Follow-up Fisher’s LSD pairwise comparisons (Fisher, 1990) revealed that participants in the distant temporal distance condition formed more favorable evaluations (*M* = 3.95, *SD* = 1.35) than those in the proximal temporal distance [*M* = 3.19, *SD* = 1.15, *d* = 0.61, 95% CI (0.29; 1.22)], or neutral condition [*M* = 3.33, *SD* = 1.21, *d* = 0.48, 95% CI (0.15; 1.08)], *p* < 0.01 and *p* < 0.05, respectively, which did not differ [*p* > 0.05, 95% CI (−0.61; 0.33)].

#### Mood Scores

Participants’ moods measured for first time were named T1 mood, and those assessed for the second time were named T2 mood. We conducted two separate one-way (distant temporal distance vs. proximal temporal distance vs. neutral) ANOVAs in which either T1 mood or T2 mood served as the dependent variable. The ANOVAs revealed that the effect of temporal distance on either T1 mood or T2 mood was not significant [T1 mood: *F* (2, 162) = 1.74, *p* > 0.05, *η*^2^ = 0.021; T2 mood: *F* (2, 162) = 2.34, *p* > 0.05, *η*^2^ = 0.028].

Overall, Study 4 confirmed that distant temporal distance indeed promoted creative thinking while ruling out the alternative explanation concerning the achievement motivation. Moreover, mood could also not be an alternative explanation for the observed effects.

## General Discussion

The results across the four studies consistently provided strong evidence for the benefit effect of distant temporal distance on creative thinking. Specially, participants in the distant temporal distance condition generated more original uses of a newspaper (Study 1), responded more correct answers on the CRAT (Study 2), designed more original and novel toys (Study 3), and had more favorable evaluation on the creative ad (Study 4). The results conceptually replicated the earlier findings obtained by Förster et al. (2004) and Chiu (2012). Furthermore, our finding (Study 4) also extended the two previous studies by ruling out an alternative explanation concerning the achievement motivation, further improving the generalization of the conclusion regarding the effect of distant temporal distance on creative thinking. The beneficial effect of distant temporal distance on creative thinking could be explained by several reasons. First, CLT proposes that distant temporal distance elicits individuals’ abstract mental representations (Liberman and Trope, 1998; Trope and Liberman, 2003). Increased abstract mental representations, in turn, expand individuals’ conceptual attention, therefore contributing to creative thinking (Förster et al., 2004; Ward et al., 2004; Friedman and Förster, 2010). Second, previous studies found that individuals’ emotional intensity in distant temporal distance conditions is lower than that in proximal temporal distance conditions (Davis et al., 2011). In other words, compared with psychological representations of proximal temporal distance, those of distant temporal distance weakens the weight of emotional awareness in cognitive processing (Davis et al., 2011). This allows individuals in distant temporal distance to experience less emotional interference in thinking processes, thus promoting the generation of creative ideas (Zhang et al., 2016). Third, due to individuals’ knowing less about the future events, their tolerance of uncertainty in predicting future events is higher than that in predicting recent events (Onay et al., 2013). Higher tolerance of uncertainty is then conductive to individuals’ creative thinking (Naveh and Erez, 2004).

We also found that promotion motivation mediated the beneficial effect of distant temporal distance on creative thinking. Specially, promotion motivation mediated the beneficial effect of distant temporal distance on the CRAT scores (Study 2) and the originality/novelty of the self-designed toy (Study 3). The findings confirmed the theoretical expectations of the ecological system model of creativity, which postulates that the effect of situational factors on individual’ creativity primarily comes indirectly *via* personal characteristics (Yeh, 2004). Individuals with a temporally distant perspective had abundant time to imagine what they would like to do or want to achieve (promotion-focused; Higgins, 1997, 2015; Liberman and Trope, 1998; Pennington and Roese, 2003; Mogilner et al., 2008). This, in turn, triggered a relatively risky and explorative processing style that were consistent with creative thinking (Derryberry and Tucker, 1994; Friedman and Förster, 2002; Mehta and Zhu, 2009; Rook, 2014). Also, this mediating mechanism could be accounted for by another theory: the Self-Determination Theory (SDT; Deci and Ryan, 2008). The theory maintains that when environments promote individuals’ fulfillment of the basic psychological needs for autonomy, individuals’ internalization of the values and stipulations of the environments will be enhanced. Thus, their autonomous motivation (which was defined as people’s acting with a full sense of choice and volition, and includes promotion motivation) will then be facilitated, therefore boosting their creative outcomes. Since distant temporal distance provided adequate temporal resource (Higgins, 1997, 2015), it shaped an environment characterized by freedom and spontaneity inherent, and thus individuals’ need for autonomy was satisfied. As a result, individuals’ autonomous motivation, including promotion motivation, was fostered, subsequently enhancing creative thinking.

Our results have several practical implications in cultivating individuals’ creativity. First, distant temporal distance exerted beneficial effects on creative thinking. Thus, when completing creative activities, individuals should imagine distant future lives for some time. Second, promotion motivation was an important mediator of the association between distant temporal distance and individuals’ creative thinking. According to Higgins (1997), when individuals consider their dreams and wishes, their promotion motivation will be induced, while when they consider their responsibilities and obligations, their prevention motivation will be induced. Accordingly, individuals can also be advised to think about their dreams and wishes before creative thinking tasks.

The present work was not without limitations that should be addressed in future research. First, only one type of temporal distance manipulation was used in the present study. Future research should include other methods to manipulate temporal distance to provide more evidence for the observed effects. Second, the samples in the present study were all undergraduates and whether such findings emerge for other samples was unknown. Third, even though the effect of distant temporal distance was replicated across four creative thinking tasks, it cannot be assumed that the results will remain similar across all creative thinking assessments, especially for the creative thinking tasks which are rooted in different theoretical backgrounds from those in the present study.

An important strength of the present research was the adoption of a special creative thinking task – the AET – in Study 4. By using this task, we verified that distant temporal distance indeed enhanced creative thinking while ruling out the alternative explanation concerning the achievement motivation. In addition, the present research, for the first time, contributed to the underlying motivation mechanism through which distant temporal distance enhanced creative thinking by reporting that promotion motivation mediated the beneficial effect of distant temporal distance on creative thinking.

## Conclusion

The current research has established nuanced, systematic, and parsimonious relationships among temporal distance, promotion motivation and creative thinking. Specifically, distant temporal distance enhanced creative thinking. Furthermore, promotion motivation mediated the beneficial effect of distant temporal distance on creative thinking.

## Data Availability Statement

The datasets using in this study are available from the corresponding author on reasonable request.

## Ethics Statement

The studies involving human participants were reviewed and approved by the ethics committee of Shandong Normal University. The patients/participants provided their written informed consent to participate in this study.

## Author Contributions

PC wrote the whole paper. JZ revised the whole paper. YQ collected data of this paper.

### Conflict of Interest

The authors declare that the research was conducted in the absence of any commercial or financial relationships that could be construed as a potential conflict of interest.
