# Supplementary material for: Distant Temporal Distance and Creative Thinking: The Mediating Role of Promotion Motivation
Source: Front Psychol. 2020 Nov 6;11:576835. doi: 10.3389/fpsyg.2020.576835 (PMC7677139; doi:10.3389/fpsyg.2020.576835)
Supplement: Supplementary file 1 [file Data_Sheet_1.doc]

**The drawings in Study 3**


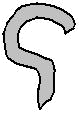

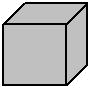

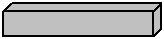

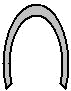


实心钩 倒u形 三维矩形 三维立方体


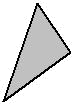

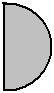

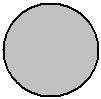

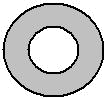


三维半球 三维球 平面环形 平面三角形


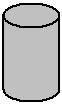

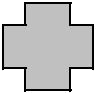

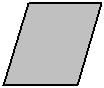


三维锥体 三维圆柱 平面十字 平面菱形


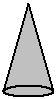

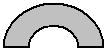

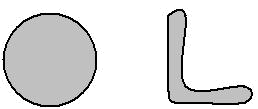


平面空心方形 三维手柄 平面圆盘 三维支架


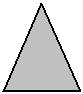

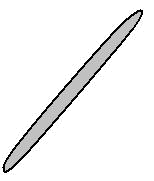

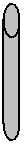


长棍 细杆 三角形 平面正方形

Note: the information in the above figure can be translated in English as follows:

The first line: 3-D Half Sphere; 3-D Sphere; Flat Ring; Flat Triangle.

The second line: 3-D Cone; 3-D Cylinder; Flat Cross; Flat Diamond.

The third line: Flat, Hollow Square; 3-D Handle; Flat Circle (disk); 3-D Bracket.

The fourth line: Solid Hook; Inverted u-shaped; 3-D Rectangular Block; 3-D Cube.

The fifth line: Long stick; Thin Pole; Triangle; Flat Square.

**The ad picture in Study 4**


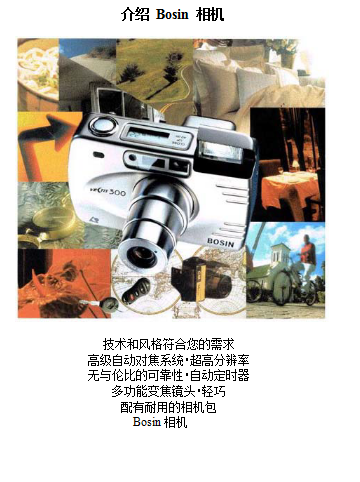


Note: the information in the above figure can be translated in English as follows:

The title: Introducing Bosin Camera.

The first line (under the picture): The Technology and Style to Match Your Needs

The second line (under the picture): Advanced autofocus system • Exceptional resolution

The third line (under the picture): Unsurpassed reliability • Self-timer

The fourth line (under the picture) Highly versatile zoom lens • Lightweight

The fifth line (under the picture): Comes with durable carrying case

The sixth line (under the picture): Bosin Camera
